# Emerging Roles of TRIM Family Proteins in Gliomas Pathogenesis

**DOI:** 10.3390/cancers14184536

**Published:** 2022-09-19

**Authors:** Angeliki-Ioanna Giannopoulou, Charalampos Xanthopoulos, Christina Piperi, Efterpi Kostareli

**Affiliations:** 1 Department of Biological Chemistry, Medical School, National and Kapodistrian University of Athens, 11527 Athens, Greece; 2 Wellcome-Wolfson Institute for Experimental Medicine, School of Medicine, Dentistry and Biomedical Sciences, Queen’s University Belfast, Belfast BT9 7B, UK

**Keywords:** gliomas, brain tumors, gliomagenesis, glioblastoma, TRIM proteins, tripartite motif, RBCC proteins

## Abstract

**Simple Summary:**

Gliomas remain challenging tumors due to their increased heterogeneity, complex molecular profile, and infiltrative phenotype that are often associated with a dismal prognosis. In a constant search for molecular changes and associated mechanisms, the TRIM protein family has emerged as an important area of investigation because of the regulation of vital cellular processes involved in brain pathophysiology that may possibly lead to brain tumor development. Herein, we discuss the diverse role of TRIM proteins in glioma progression, aiming to detect potential targets for future intervention.

**Abstract:**

Gliomas encompass a vast category of CNS tumors affecting both adults and children. Treatment and diagnosis are often impeded due to intratumor heterogeneity and the aggressive nature of the more malignant forms. It is therefore essential to elucidate the molecular mechanisms and explore the intracellular signaling pathways underlying tumor pathology to provide more promising diagnostic, prognostic, and therapeutic tools for gliomas. The tripartite motif-containing (TRIM) superfamily of proteins plays a key role in many physiological cellular processes, including brain development and function. Emerging evidence supports the association of TRIMs with a wide variety of cancers, exhibiting both an oncogenic as well as a tumor suppressive role depending on cancer type. In this review, we provide evidence of the pivotal role of TRIM proteins in gliomagenesis and exploit their potential as prognostic biomarkers and therapeutic targets.

## 1. Introduction

Gliomas represent a group of central nervous system (CNS) neoplasms which account for the majority of primary brain tumors both in adults and children [1]. Although they have been named after glial cells, their origin is still rather ambiguous. Several lines of evidence suggest that they are derived from neural stem cells (NSCs) or oligodendrocyte progenitor cells (OPCs), normal glial cells, or other cell types [2]. Based on the recent elucidation of histopathological and molecular features characterizing gliomas, the latest 2021 WHO classification of tumors of the CNS encompasses distinct glioma families, including adult-type diffuse gliomas, pediatric-type diffuse low-grade gliomas, pediatric-type diffuse high-grade gliomas, and circumscribed astrocytic gliomas [3]. Each glioma family contains several tumor types, using a grading system of Arabic numerals (1–4). More specifically, the adult-type diffuse gliomas include the astrocytomas with IDH-mutant, the oligodendrogliomas with IDH-mutant and 1p/19q-codeleted, as well as the glioblastomas with IDH-wildtype. The pediatric-type diffuse low-grade gliomas include the diffuse astrocytomas with MYB- or MYBL1-altered, the angiocentric gliomas, the polymorphous low-grade neuroepithelial tumors of the young, and the diffuse low-grade gliomas with MAPK pathway-altered. Furthermore, the pediatric-type diffuse high-grade gliomas include the diffuse midline gliomas with H3 K27-altered, the diffuse hemispheric gliomas with H3 G34-mutant, the diffuse pediatric-type high-grade gliomas with H3-wildtype and IDH-wildtype and the infant-type hemispheric gliomas. The circumscribed astrocytic gliomas encompass the pilocytic astrocytomas, the high-grade astrocytomas with piloid features, the pleomorphic xanthoastrocytomas, the subependymal giant cell astrocytomas, the chordoid gliomas, and the astroblastomas with MN1-altered [4]. Moreover, the new WHO classification has defined ten specific ependymoma tumor types characterized based on their specific location and molecular characteristics [3].

The diagnosis of different glioma tumor types is highly based on their molecular peculiarities. Adult-type diffuse gliomas are graded and characterized by the presence or lack of IDH mutations, chromosome 7 gain and/or loss of chromosome 10, EGFR amplification, TERT promoter mutations, CDKN2A/B deletion, PTEN mutations or deletion, TP53 and ATRX mutations, MDM2 or MDM4 amplification, BRAFV600E mutations, and MGMT promoter methylation. Pediatric-type diffuse low-grade gliomas are characterized by mitogen-activated protein kinase (MAPK) pathway activation and by lack of IDH or histone mutations. Aberrant MAPK signaling may result from BRAF V600E mutations, NTRK alterations, and FGFR/2/3 or MYB/MYBL1 fusions. Pediatric-type diffuse high-grade gliomas are mostly characterized by the presence or absence of histone 3 (H3) mutations, such as H3K27M and H3G34V/R. However, RTK-activating fusions, MYCN amplification, and EGFR, PDGFRA, TP53, NF1, or TERT mutations are also present among the different types. Circumscribed astrocytic gliomas may carry *PRKCA, MN1*, MAPK cascade-activating alterations, and *IDH* mutations [4].

Although circumscribed gliomas are considered benign and can be removed by complete surgical resection, diffuse types are more aggressive and require further treatment following surgical resection with limited options. To improve treatment results and diagnosis of gliomas, a continuous effort to understand the molecular mechanisms and underlying signaling pathways involved in gliomagenesis is mandatory [5]. Current studies have shown that activation of key signaling pathways is regulated by multiple molecules, including the tripartite motif-containing (TRIM) protein family. This is one of the most prominent families of RING domain-containing E3 ligases which mediate post-translational modifications and regulate a wide range of cellular processes [6]. In this review, we discuss the structural and functional diversity and the biological impact of TRIM proteins in brain physiology, highlighting their pivotal role in the development and progression of brain tumors.

## 2. TRIM Proteins: An Overview of Structure and Function

**Figure 1:**
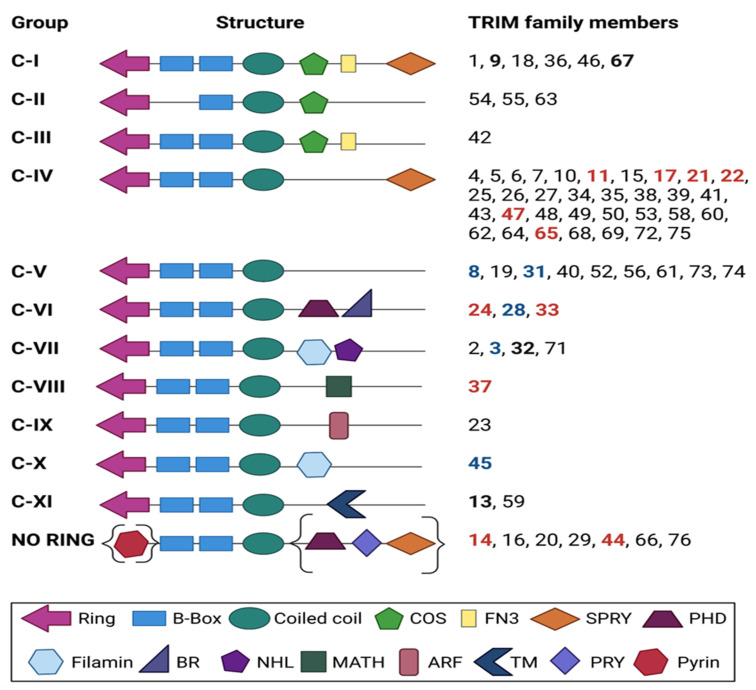
Classification of human TRIM proteins based on the nature of their C-terminal domains. The TRIM protein family is composed of 11 subfamilies, from CI to CXI, and one unclassified group of RING-less TRIM proteins. Individual proteins involved in brain physiology and tumors are marked with black and red, respectively, while the ones reported in both cases are shown in blue (created by BioRender.com (2022)).

TRIM (tripartite motif-containing) family proteins, also known as RBCC proteins (from the initials of RING-B-box-Coiled-Coil regions), bear a characteristic N-terminal tripartite (RBCC) motif. This motif is comprised in most cases of a RING domain, either one or two B-boxes (B1 and B2), and a coiled-coil (CC) domain, followed by a C-terminal domain (Figure 1). Based on the presence of a RING domain, most TRIMs are defined as E3 ligases. E3 ligases are responsible for substrate recognition [7,8,9,10] by directly binding to and activating E2 conjugating enzymes [11,12], which mediate the attachment of proteins to ubiquitin, SUMO, ISG15, or NEDD8 [13], therefore contributing to the biological flexibility of numerous TRIM proteins [9]. E3 ligase-independent biological roles of TRIM proteins have also been identified, including RNA-binding [14]. TRIM proteins can also form homopolymers and heteropolymers with each other through their coiled-coil domain, while the B-box domains are designated as a universal domain in TRIMs [15]. To date, more than 70 TRIM family members have been reported in humans and mice [16]. Their classification into subfamilies (I to XI) is based on differences in the C-terminal domain [16,17,18] (Figure 1). About 60% of TRIMs contain the PRY-SPRY domain (also known as B30.2), which confers to protein–protein interactions and RNA binding [19,20], along with the NHL domain [21]. The C-terminal subgroup one signature (COS) domain is necessary for microtubule binding [16], while the plant homeodomain (PHD) with the adjacent bromodomain (BRD), provides DNA-binding properties and confers transcriptional regulation [22]. For example, TRIM24 was shown to bear epigenetic reader functions via the interaction of its tandem PHD-bromodomain with chromatin [23]. Another common domain identified among TRIM proteins is the ADP ribosylation factor-like (ARF) domain, which regulates intracellular trafficking due to its GTP hydrolysis activity [24]. The fibronectin type III motif (FN3) is primarily found in cell surface proteins, all of which are involved in a molecule recognition [25]. The filamin domain possesses an immunoglobulin-like structure which is involved in mRNA regulation for TRIM-NHL proteins [21]. Finally, the meprin and tumor-necrosis factor receptor-associated factor homology (MATH) domain is involved in receptor binding and oligomerization [18]. Notably, some TRIM proteins do not contain a RING (Figure 1), such as the PRY-SPRY motif-containing TRIM14 and TRIM16 or the PHD- and BRD-containing TRIM66 [26] (Figure 1).

Bearing these structural features, it is no surprise that TRIM proteins are involved in a broad range of biological processes, including transcriptional regulation, DNA repair, cell proliferation, apoptosis, autophagy, metabolic and immune signaling, stem cell differentiation, and neurogenesis [13,27,28,29]. A single TRIM protein can play multifaced roles within a cell. For instance, TRIM28 (also called KAP1) can mediate DNA damage response, maintain stem cell pluripotency, induce autophagy [30], and also act as a transcription regulator. It can interact with chromatin and confer transcriptional repression by localizing in Lamina-associated domains, or function as a critical metabolic regulator through its E3 ubiquitin ligase function [31,32]. The transcriptional regulation properties of TRIM28 concern both activation and repression of essential transcriptional programs and has raised considerable interest across disciplines and conditions. Hitherto, the underlying mechanisms of this functional duality have not been fully elucidated and are highly required for planning translational approaches and applicability in a clinical context [33].

About half of all TRIM proteins have been reported to contribute to autophagy [27,34,35,36,37] by mediating binding to components of the autophagy machinery and/or to receptors that recognize ubiquitinated autophagy cargo and forming the so-called TRIMosomes. TRIM-mediated autophagy has been shown to target viral components and to be critically involved in innate immunity [24,38]. The functional role of TRIM proteins during infection has been highlighted by numerous studies and review articles. TRIMs promote host defense against viral infection, whereas viruses possess adaptation strategies to hijack TRIM-mediated defense mechanisms [39,40]. Apparently, the broad range of TRIM functions are critical not only at a cellular level but also at a system level and are tightly linked to human health and disease.

**Figure 2:**
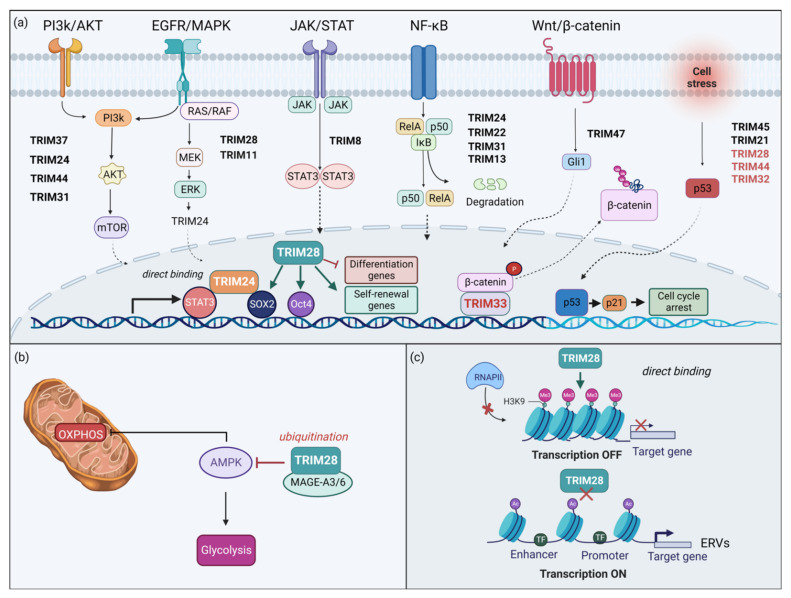
Representation of the multifaced functional roles of TRIM proteins within a brain tumor cell. (**a**) Summary of TRIM proteins and their involvement in critical signaling pathways and gene regulation. Their contribution to activation (positively correlated TRIMs) is marked with bold black letters, while the ones leading to downregulation of the pathway (negatively correlated TRIMs) are shown with red. (**b**) Characteristic example of TRIM28 oncogenic mechanism of function leading to autophagy inactivation and mitochondrial dysfunction through AMPK inhibition in a glioma cell. (**c**) TRIM28 epigenetic mechanism of action for transcription regulation in neuronal progenitor cells. TRIM28 plays a key role in controlling neuronal differentiation processes by establishing local heterochromatin with H3K9me3 enrichment to transposable elements and their target genes which are responsible for neuronal proliferation. Loss of TRIM28 leads to upregulation of the ERV-mediated transcriptional network (created by BioRender.com (2022)).

Besides acting as an antiviral host defense, a large number of TRIM proteins have been reported to exert oncogenic or tumor suppressive potential in solid tumors and blood cancers [41,42]. The emerging significance of TRIM proteins in various malignancies arises not only from key mechanistic insights into tumorigenesis but also from the important translational potential. As for the latter, TRIM-targeted therapies in cancer are gaining momentum over the past years, such as the TRIM8-targeting approaches for chemo-resistant colorectal cancer [43] or the TRIM24-targeting options for glioblastoma [44]. However, as mentioned above, pharmacological targeting of TRIMs and the transition from bench to the clinic is very much dependent on elucidating the functional role of each TRIM of interest per cancer entity. Functional duality (as an oncogene or a tumor suppressor) can represent a pure challenge for therapeutic interventions and is often depicted in the complexity of molecular signaling cascades [45,46,47] that TRIM regulate or can be regulated by (Figure 2).

TRIM proteins are also involved in numerous non-cancerous human pathologies, including inflammatory and autoimmune disorders, as they possess key roles in immune-related pathways. For instance, TRIM21 is involved in GSDMD-mediated pyroptosis and has been suggested as a target for controlling inflammation and inflammatory-related disorders [48]. TRIM21 was also reported to contribute to abnormal cytokine production in Systemic lupus erythematosus (SLE) [49]. A recent study describing exciting crosstalk between epigenetic regulation and optineurin (OPTN)-autophagy was identified in the context of inflammation. TRIM14 was shown to act as an epigenetic regulator that reduces histone H3K9 trimethylation by inhibiting the OPTN-mediated selective autophagic degradation of the histone demethylase KDM4D. Additionally, TRIM14-deficient dendritic cells led to impaired KDM4D-directed proinflammatory cytokines and offered protection from autoimmune inflammation [50].

Finally, it is worth noting that various TRIM proteins have been implicated in metabolic disorders, ranging from obesity (i.e., TRIM23, TRIM25, TRIM28, and TRIM67) [51,52] to diabetes (i.e., TRIM7, TRIM27, TRIM32, and TRIM72), [53] as well as cardiovascular diseases [54]. The muscle-specific TRIM called Mitsugumin 53 (MG53 or TRIM72) is leading research attention in this field, and it has been shown to negatively regulate myogenesis and promote diabetic as well as cardiovascular complications. Importantly, TRIM72 induces insulin resistance by mediating (as an E3 ligase) the degradation of the insulin receptor and insulin receptor substrate-1 (IRS1) [55,56].

This broad involvement of TRIM proteins in various physiological processes and diseases further highlights their versatile functionality and clinical significance arising from their multifaceted molecular roles: from regulating numerous genes and proteins within the cell at the transcriptional level (epigenetic reader, co-transcription factor, and chromatin accessibility: nuclear functions) to the posttranslational level regulation (E3 ligase, protein degradation, and protein subcellular localization: cytoplasmic functions) (Figure 2). Altogether, TRIM family proteins’ structure (Figure 1) is well mirrored on the remarkable range of functions, such as their involvement in PI3K/AKT, JAK/STAT, NF-κB, and EGFR/MAPK (Figure 2a) in addition to their role in mitochondrial function and metabolism (Figure 2b) and their critical epigenetic functions (Figure 2c).

## 3. TRIM Proteins and the Nervous System: Insights into Brain Physiology and Pathophysiology

Since TRIM proteins are highly involved in stem cell regulation and differentiation, they play essential roles in the function of neurons and the physiology of the nervous system. Research efforts aiming at shedding light on TRIM function in normal and pathological conditions in the nervous system have attracted increasing interest over the past decade. The most recent and prominent cases of the role of TRIM proteins for brain physiology and pathophysiology (besides brain cancer) are discussed in the following section.

### 3.1. Healthy Brain

TRIM28—along with TRIM24, TRIM33, and TRIM66—is a member of the TIF1 family of chromatin-binding proteins (TIF1: transcriptional intermediary factor 1) with critical roles in the regulation of stem cell and brain development [57]. TRIM28 has been identified as one of the main epigenetic modifiers that controls the transition between somatic and pluripotent states within a cell (Table 1). The underlying mechanism involves the generation of a heterochromatin environment with H3K9me3 marks, which leads to silencing of endogenous retroviruses (ERVs) and blocking the expression of specific genes. Ectopic expression of OCT4, SOX2, KLF4, and c-MYC (OSKM) is known to be used for cell reprogramming and formation of pluripotent stem cells. When this expression of OSKM was accompanied by *TRIM28* knockdown, an increased expression of genes around H3K9me3 was detected, which enhanced the reprogramming efficiency [58]. A key study has shown that TRIM28 regulates the transcription of neural progenitor cells (NPCs) by silencing ERVs. This affects transcriptional dynamics by activation of nearby genes and expression of long non-coding RNAs (lncRNAs). Moreover, lack of TRIM28 was shown to lead to overexpression of ERV groups IAP1 and MMERVK10C. The reported TRIM28-mediated gene regulation is of high clinical significance, offering a link between ERVs and brain disorders [59]. Brattas et al. expanded upon that work and reported a TRIM28-dependent gene regulatory network based on ERVs, which is implicated in the control of gene expression for transcripts that are important for brain development [60]. TRIM28 interaction with Paupar lncRNA was further shown to affect target gene expression for neuronal differentiation. Specifically, Paupar was demonstrated to promote TRIM28 chromatin occupancy and H3K9me3 enrichment at a subset of distal targets, forming a ribonucleoprotein complex containing Paupar, TRIM28 and the PAX6 transcription factor. The association of the complex in chromatin was identified within the regulatory region of shared target genes, critical for neural cell proliferation and function [61]. Interestingly, TRIM28 was identified as a master regulator for gene expression in the mammalian brain by binding thousands of transposable elements (TEs) in NPCs and influencing nearby gene expression [62].

TRIM11, a member of the largest family group (Group IV, Figure 1) plays a pivotal role in modulating cortical neurogenesis by altering Pax6 transcription factor levels [63]. Specifically, it was shown that TRIM11 binds the neurogenic factor Pax6 for ubiquitous degradation and that TRIM11 overexpression is followed by decreased Pax6 levels, while lack of the protein increases the insoluble Pax6 levels, leading to apoptotic events in the developing brain [63] (Table 1).

A role for TRIM3 (Family Group C-VII, Figure 1) in regulating post synaptic density (PSD) proteins composition and dendritic spine morphology has been reported by Hung and colleagues (Table 1). They showed that TRIM3 is one of the key ubiquitin-related proteins in actively stimulated neurons, by recognizing the PSD scaffold GKAP/SAPAP1 for degradation. Interestingly, RNAi assays for TRIM3 at post synaptic sites increased the levels of GKAP/SAPAP1 and resulted in dendritic spine head enlargement [64].

TRIM45, the only member of Group C-X, is critical for diencephalon and eye development at the early stages of zebrafish morphogenesis (Table 1). Knockdown of *TRIM45* led to a reduction of both tissues’ size at twenty-four hours post-fertilization and affected the spatial distribution of *olig2* and *rx1/rx3* marker genes in the diencephalon, retina, and optic primordia [65].

Another member of TRIM family with an implicated role in brain physiology is TRIM67 (Group C-I, Figure 1). Boyer et al. identified that TRIM67 is critical for proper mammalian brain development, cognitive ability, and social behavior. The researchers generated a knockout mouse and used a suite of behavioral assays to clarify the physiological importance of the specific ubiquitin ligase. *TRIM67-*deficient showed defects in the development of specific brain regions and neural functions such as spatial memory, motor function, sociability and sensorimotor gating [66] (Table 1). One year later, the same group reported that the ubiquitin ligase affects the development of specific axon tracts and filopodia dynamics by interfering and competing with another E3 ligase and Group C-I member, named TRIM9 [67]. Focusing on Group C-I members TRIM9/TRIM67 interplay in neuronal development, they identified that both are essential for appropriate morphogenesis of cortical and hippocampal neurons and responses to the axon guidance cue netrin-1 (Table 1). Furthermore, they evaluated the mechanistic effects of their interaction, by performing an unbiased proximity-dependent biotin identification (BioID) approach and reported a restricted list of co-interactors, which showed dynamic co-localization with both TRIM proteins at the axonal periphery, including at the tips of the filopodia [68].

### 3.2. Pathological Conditions-Injured Brain

Inflammatory challenges posed by traumatic brain and spinal cord injury, infections, toxins exposure, microbes, neurodegenerative disease, or aging can activate the brain’s innate immune system leading to chronic inflammatory responses of the nervous tissues (referred as neuroinflammation). Chronic inflammation involves the sustained activation of glial cells (microglia and astrocytes) and recruitment of other immune cells into the brain which secrete reactive oxygen species, growth factors and cytokines, compromising the blood–brain barrier. Evaluating the role of TRIM9 in pathological conditions in the brain, which is a key driver of brain injury, Zen et al. identified that the ligase function is critical for resolving NF-κB-dependent neuroinflammation and, therefore, alleviation of stroke damage. *TRIM9*-knockout mice were reported to be more vulnerable to ischemia than wild-type, while systemic administration of a recombinant TRIM9 adeno-associated virus effectively restricted inflammation [69]. TRIM32 (Group C-VII) was also reported to have a key role in a neuropathological condition, and specifically in cerebral ischemia reperfusion injuries (Table 1). TRIM32 was overexpressed in hippocampal neurons subjected to oxygen-glucose deprivation/reperfusion (OGD/R) as compared to normoxia conditions. Interestingly, knockdown of *TRIM32* led to a Nrf2 pathway-dependent protection of hippocampal neurons from OGD/R-induced oxidative injury [70].

Zen and colleagues suggested TRIM31 (Group C-V) as a potential target for ischemic stroke therapy (Table 1). Their findings showed that TRIM31 triggered cerebral ischemic injury by ubiquitous degradation of the apoptosis regulator TIGAR, resulting in ROS, deregulation and mitochondrial dysfunction after brain ischemia [71]. Another potential regulator during ischemic injury is TRIM45 (Group C-X), as reported more recently. Specifically, it was demonstrated that TRIM45 regulates cerebral I/R injury by activating NF-κB signaling in microglia through interaction with TAB2, consequently leading to neuronal apoptosis. Knockdown of *TRIM45* has proved to provide striking results for the above effects by inhibiting the inflammatory response [72].

Another interesting study aimed to shed light on the role of TRIM13 (Group C-XI, Figure 1) in brain injury, focusing on high fat diet (HFD)-induced CNS damage (Table 1). Brain-specific deletion of *TRIM13* in mice promoted HFD-induced metabolic disorder, hypothalamic insulin resistance and systematic inflammatory response. In vitro analysis of *TRIM13* knockout glial cells showed an enhanced palmitate (PAL)-induced inflammatory response by accelerating the NF-κB signal, which then contributed to the insulin resistance in the isolated primary neuron [73].

TRIM proteins also play a role in brain disorders such as Schizophrenia and Alzheimer’s disease (Table 1). For instance, TRIM11 was identified to negatively regulate the peptide Humanin, which has neuroprotective roles against Alzheimer’s. The deletion of the B30.2 domain of TRIM11 or alteration of the RING finger domain sequence blocked the interaction with Humanin [74]. TRIM8 has been suggested to be one of the key genes for schizophrenia etiology by regulating neural development and synaptic functions. From a mechanistic point of view, it was shown that the transcription factor POU3F2 is involved, since TRIM8 expression levels were induced upon POU3F2 binding to a schizophrenia-associated SNP, located within the *TRIM8* promoter area [75]. Interestingly, de novo mutations on the highly conserved C-terminus section of *TRIM8* have been linked with four cases of patients who developed Epileptic Encephalopathy (EE) [76,77].

TRIM3 has been also involved in the molecular mechanisms underlying neuronal disorders (Table 1). Work with in vivo and in vitro Parkinson’s disease (PD) models, showed that TRIM3 is involved in the reduction of an apoptotic phenotype in PD cells through the PI3K/Akt pathway [78]. Mutations in *TRIM32* have been associated with a rare autosomal recessive degenerative myopathy called Limb–Girdle Muscular Dystrophy R8 (LGMDR8) [79], which is characterized by important neurological defects (Table 1). Experimental work with *TRIM32* knockout mice showed that a lack of TRIM32 negatively impacts the concentration of neurofilament proteins in the brain and the diameter of the motor axon. Additionally, in neural stem cells (NSCs), TRIM32 distributes asymmetrically upon cell division and promotes cell differentiation via translocating into the nucleus and targeting of c-MYC for degradation [80].

It becomes apparent, that the importance of TRIM proteins for both physiology and pathology of the neuron, the brain and the nervous system has been to-date only fragmentarily unraveled. A more systematic view across conditions with multidisciplinary work is required for an in-depth understanding of the impact of TRIM proteins’ functional versality on brain and nervous system in health and disease.

**Table 1:** TRIM protein family members implicated in brain physiology and related pathologies.

Group	TRIM	Role in Brain Physiology	Role in Brain Pathologies	References
C-I	TRIM67	mammalian brain development cognitive ability and social behavior morphogenesis of cortical and hippocampal neurons response to the axon guidance cue netrin-1	-	[66,67,68]
C-I	TRIM9	brain-specific ubiquitin (Ub) ligase regulates netrin-dependent axon guidance and morphogenesis of cortical and hippocampal neurons response to the axon guidance cue netrin-1	regulation of NF-κB-dependent neuroinflammation highly expressed in the peri-infarct areas shortly after ischemic insults in mice promotes recovery and repair after brain injury in mice	[67,68]
C-IV	TRIM11	involved in cortical neurogenesis	implication in Alzheimer’s development	[63,74]
C-V	TRIM8	controls neural development and synaptic functions	role in schizophrenia etiology de novo TRIM8 mutations involved in epileptic encephalopathy	[75,76]
C-V	TRIM31	regulates TP53-induced glycolysis and apoptosis regulator (TIGAR) in neurons	triggers cerebral ischemic injury through enhanced reactive oxygen species (ROS) production	[71]
C-VI	TRIM28	epigenetic regulator of gene transcription reprogramming pluripotent-somatic cell state transition neural cell differentiation, proliferation, and function	activation of human endogenous retroviruses (HERVs) related to autism spectrum disorder (ASD)	[58,59,60,61,62]
C-VII	TRIM3	involved in dendritic spine morphology by regulating PSD proteins composition	regulation of apoptosis in Parkinson’s disease (PD) cell model via PI3K/AKT signalling pathway activation	[64,78]
C-VII	TRIM32	promotes cell differentiation in NSCs involved in neural regeneration indirect regulation of proteome in the brain and motor axon formation	involved in cerebral ischemia reperfusion injury TRIM32 mutations implicated in Limb–Girdle Muscular Dystrophy R8 (LGMDR8)	[70]
C-X	TRIM45	eye development and diencephalon development	regulation of cerebral ischemia reperfusion injury	[65,72]
C-XI	TRIM13	highly expressed in the brains of human adult and embryonic tissues regulates NF-κB signaling in microglia	regulation of neuroinflammation during ischemic injury involved in cerebral ischemia and reperfusion injury process in mice	[72]

## 4. TRIM Proteins in Brain Tumors

The versatile role of TRIM proteins is illustrated by their involvement in a broad range of molecular pathways and biological processes. Many TRIMs have been linked to carcinogenesis, and changes in their expression have been strongly correlated with the cancer type or stage and disease outcome [36]. An increasing number of TRIMs are involved in glioma development and progression (Table 2) with oncogenic, tumor suppressor or a dual role targeting one or more proteins and pathways at the cytoplasm, and/or exerting their gene regulatory/epigenetic function in the nucleus at a genome-wide level, thereby affecting multiple genes (Figure 2). For instance, an oncogenic role for TRIM37 in glioma progression has been reported since TRIM37 targets proliferation, migration/invasion, and the epithelial–mesenchymal transition (EMT) via the regulation of the PI3K/Akt pathway [81] (Figure 2a and Table 2). TRIM28 is overexpressed in gliomas, and its expression inversely correlates with overall survival and progression-free survival [82]. TRIM24 overexpression is characteristic of gliomas and is required for EGFR activation and for STAT3 recruitment and stabilization, which is important for exerting its oncogenic potential [83,84] (Figure 2a and Table 2). At the same time, a tumor suppressive role has been suggested for TRIM45 as it was found to interact with p53 and stabilize it through the K63-linked ubiquitination, thus impairing GBM proliferation and tumorigenicity [85] (Figure 2a and Table 2).

### 4.1. Family Group C-IV: TRIM11, TRIM17, TRIM21, TRIM22, TRIM47 and TRIM65

The TRIM proteins of Group C-IV, the larger family group, are characterized by the lack of COS, FN3, PHD, and NHL domains (Figure 1). One of the early studies identified TRIM11 as being upregulated in high-grade gliomas (HGG) and glioma-derived stem-like cells (GSCs), leading to a more aggressive glioma phenotype [86]. TRIM11 has been suggested as a poor prognostic marker in gliomas, since HGG patients with lower levels of TRIM11 exhibited prolonged survival compared to those with a higher TRIM11 expression. TRIM11 can also serve as a superior biomarker for GSC detection compared to CD133 and nestin. Knockdown of *TRIM11* inhibited glioma cell proliferation, migration, and invasion, whereas low levels of TRIM11 resulted in downregulation of EGFR, p-c-Raf, p-MEK1/2, and p-44/42MAPK. *TRIM11* silencing led to a decrease in HB-EGF (heparin-binding EGF-like growth factor) and CCND1 (Cyclin D1) expression, while *EGFR* levels remained stable. Furthermore, overexpression experiments suggest that TRIM11 exerts its oncogenic function in gliomas via EGFR/MAPK signaling (Figure 2a) and most likely without involvement of the PI3K/Akt pathway [86].

Xiao et al. identified a TRIM-based gene expression signature which could serve as a prognostic biomarker for overall survival. High expression of TRIM13, TRIM17, and TRIM8 was indicative of the high-risk group, while TRIM24, TRIM14, TRIM29, TRIM59, and TRIM38 were indicative of the low-risk group. Importantly, TRIM17 was downregulated in gliomas compared to normal brain tissue, and its expression was inversely correlated with tumor grade. Functional assays on glioma cell lines supported a tumor suppressive role for TRIM17 involving suppression of cell proliferation [87].

TRIM21 has been recently shown to serve as a poor prognosis marker in gliomas. It was reported to be highly expressed in glioma cases compared to a normal brain and its levels were correlated with the malignancy grade. Elevated TRIM21 levels were significantly associated with a poor prognosis in all glioma types, including HGGs and GBM, suggesting an oncogenic role for this TRIM protein. Gain-of-function or loss-of-function assays in glioma cell lines indicated that TRIM21 mediates cell proliferation and migration. Experiments in xenograft murine models confirmed the oncogenic potential of TRIM21 since mice injected with glioma cells overexpressing TRIM21 or depleted of TRIM21, exhibited decreased and increased OS, respectively, compared to control mice. In addition, differential expression analysis between control and *TRIM21-*silenced glioma cells revealed numerous genes involved in cellular senescence pathways. *TRIM21* knockdown resulted in the downregulation of TP53 and CDKN1, whereas its overexpression showed opposite effects. TRIM21-mediated cellular senescence in glioma cells was shown to involve the p53/p21 pathway, as a potential mechanism for glioma progression. The link between temozolomide (TMZ) resistance, aberrant p53 function, and TRIM21 was further investigated. TRIM21 mRNA levels were elevated in GBM patients with IDH1^wt^ or non-G-CIMP, in contrast with those carrying IDH1 mutations or G-CIMP. Likewise, TRIM21 expression followed a similar pattern in patients with unmethylated and methylated MGMT. In vitro work suggests that TRIM21 promotes glioma cell resistance to TMZ. Therefore, TRIM21-mediated TMZ resistance could justify the poor prognosis of glioma patients with a high TRIM21 expression [88].

TRIM22 was shown to confer GBM cell proliferation in vitro and tumor growth in vivo, possibly through TRIM22 E3 ubiquitin ligase activity. Moreover, knockdown of *TRIM22* was linked to reduced NF-κB signaling and increased half-life of the IκBα protein, possibly attributed to reduced proteasomal degradation and augmented stabilization of IκBα protein with a lack of TRIM22 ligase activity. Ji et al. also showed that TRIM22 mediates NF-κB signaling by activation of the IKK complex, since decreased TRIM22 expression correlated positively with the phosphorylation of IKKα/β (Ser176/180), IκBα (Ser32/36), P65, and K63 ubiquitination levels of IKKγ. Additionally, the study used a constitutively stable mutant form of ΙκΒα to demonstrate that the growth-promoting properties of TRIM22 relied upon ΙκΒα function both in vitro and in vivo. Interestingly, TRIM22 expression was further increased in primary HGGs samples compared to LGGs, while it was almost absent in normal tissues. In addition, high levels of TRIM22 were linked to IDH1-wild type and ATRX-wild type gliomas. In agreement with the above effects of TRIM22 expression in gliomas, transfected mice with *TRIM22*-knockdown primary GBM cells led to a reduction of tumor volume and a longer survival period [89].

TRIM47 expression was found to be elevated in glioma specimens compared to normal brain tissues. Knockdown experiments in TRIM47-highly expressing glioma cell lines, attenuated the proliferative, invasive, and migratory potential of these cells and decreased the expression of EMT markers, while an abrogation of tumor growth was observed in vivo. Moreover, it was shown that the effects of *TRIM47* knockdown were mediated by the Wnt/b-catenin pathway. While downregulation of TRIM47 lowered the expression levels of β-catenin, c-MYC, and cyclin D1, exogenous activation of Wnt/β-catenin signaling (Figure 2a) abolished the previous effects of TRIM47 knockdown observed in glioma cells [90]. Furthermore, Ji et al. showed that TRIM47 expression was higher in GBM and low-grade glioma specimens compared to normal brain and also correlated positively with the malignancy grade [91]. TRIM47 expression was shown to have a prognostic value in gliomas as a marker for poorer overall survival (OS). In addition, *TRIM47* knockdown hampered the proliferative, invasive, and migratory potential of glioma cells in line with previous work by Chen et al. [90,91].

Hu et al. suggested a mechanism that implicated TRIM65 in glioma progression through the long noncoding RNA (LncRNA) LINC01857. LINC01857 protein levels were elevated in glioma tissues compared to normal tissues and correlated with glioma grade. Patients with high LINC01857 expression exhibited poorer survival. Upregulation of LINC01857 was reported in glioma cell lines and was shown to induce glioma cell proliferation, migration, and invasion. LINC01857 also functions as a sponge for miR-1281, which expression is decreased in glioma tissues. TRIM65 mRNA is targeted by miR-1281, and LINC01857 induces TRIM65 expression in gliomas by restraining miR-1281. Therefore, LINC01857 is believed to promote glioma progression and tumor growth via regulation of the miR-1281/TRIM65 axis [92].

### 4.2. Family Group C-V: TRIM8 and TRIM31

TRIM8 is very frequently deleted in GBMs. This finding is possibly attributed to the TRIM8 location on chromosome 10q24.3, where deletions commonly occur in *IDH*^wt^ GBMs. Nevertheless, TRIM8 protein levels in GBM tissues and cell lines were similar to their normal counterparts. Interestingly, TRIM8 was primarily located in the cytoplasm of normal brain neurons, whereas in GBM, TRIM8 was predominantly present in the nucleus of neoplastic cells. Moreover, TRIM8 expression in GBM showed a positive correlation with that of known stemness markers, including STAT3, SOX2, NESTIN, OLIG2, NANOG, and BMI. Overexpression of TRIM8 in GBM neurosphere cell lines induced expression levels of stemness mediators, such as CD133, NESTIN, Sox2, and c-MYC. In addition, overexpression of TRIM8 in patient-derived GBM cells resulted in a stemness phenotype, whereas *TRIM8*-knockdown in GBM neurosphere cells reversed stemness and promoted cell differentiation. A positive feedback loop between TRIM8 and STAT3 was reported to mediate GBM neurosphere stemness. TRIM8 was found to suppress PIAS3 protein levels by mediating its ubiquitination and proteasomal degradation, which ultimately led to STAT3 upregulation (Figure 2a). However, STAT3 promoted TRIM8 expression either directly or indirectly through c-MYC and OCT1, since binding sites for all three transcription factors were detected on the TRIM8 promoter [93].

TRIM31 was overexpressed in glioma tissues and cell lines compared to normal counterparts. Overexpression of TRIM31 in glioma cell lines enhanced cell proliferation and invasiveness whereas TRIM31 silencing impeded such abilities. Furthermore, elevated TRIM31 levels were associated with high activity of the NF-κB signaling pathway (Figure 2a). TRIM31 was shown to induce p65 translocation to the nucleus, and subsequent phosphorylation of IκBα, leading ultimately to the upregulation of numerous genes involved in tumor proliferation, migration, and invasion, such as *BCL2L1*, *Snail*, *MYC*, *MMP9*, *MMP13*, *CXCL5*, *TWIST1*, and *CCND1.* Inhibition of the NF-κB pathway abrogated the effects of TRIM31 overexpression [94]. Moreover, Shi et al. reported that TRIM31 expression was increased in high-grade gliomas (hGGs) compared to normal samples and pointed out its expression as an independent prognostic factor for poor prognosis of glioma patients. In addition, increased TRIM31 expression was associated with elevated p-Akt (S473), PCNA and E-cadherin expression. By using Akt agonists in *TRIM31*-silenced cells or Akt antagonists in TRIM31-overexpressing cells, it was shown that TRIM31 mediates tumorigenesis through Akt signaling [95] (Figure 2a).

### 4.3. Family Group C-VI: TRIM24, TRIM28, and TRIM33

Overexpression of TRIM24 (also known as TIF-1α) has been reported in high-grade gliomas (hGGs), such as anaplastic astrocytoma and GBM, compared to less malignant glial tumors and normal brain tissue. Increasing levels of TRIM24 protein levels have been detected upon disease recurrence in GBM, and TRIM24 has been suggested as a prognostic marker of adverse clinical outcomes. The oncogenic potential of TRIM24 has been linked to the promotion of the GBM growth via the PI3K/Akt signaling axis (Figure 2a) by affecting Akt phosphorylation and regulating PI3KCA through the interaction of its promoter with the PHD–Bromodomain of TRIM24. Knockdown of *TRIM24* was shown to revert chemoresistance to TMZ, and TRIM24 downregulation was shown to increase TMZ sensitivity by attenuating NF-κB activity. In this way, NF-κB binding to the O6-methylguanine-DNA methyltransferase *(MGMT)* promoter and subsequent MGMT expression is hindered. A firm correlation of TRIM24 expression with treatment outcomes has been demonstrated, as chemotherapy-treated patients with low TRIM24 expression exhibited improved overall survival (OS) and progression-free survival (PFS) compared to treated patients expressing high levels of TRIM24, as well as untreated patients of diverse TRIM24 status [84].

The expression of the EGFR mutant variant EGFRvIII, which is commonly found in GBM, has been shown to reinforce the expression of the histone H3 lysine 23 acetylation (H3K23ac) mark through its kinase activity in GBM cell lines. Lv et al. demonstrated that active EGFR signaling in GBM cells leads to TRIM24 upregulation and enhancement of the interaction of TRIM24 bromodomain with H3K27ac marks. The TRIM24/H3K27ac interaction is essential for the EGFR/EGFRvIII-dependent gliomagenesis. In this context, TRIM24 acts as a transcriptional co-activator of STAT3 (Figure 2a). *TRIM24* promoter activity is enhanced by STAT3 in EGFR/EGFRvIII active GBM cells and TRIM24 is acting as a co-factor for transcriptional regulation of STAT3-target genes, such as Inhibitor of DNA binding 1 *(ID1)*. It was found that arginine 193 (R193) and lysine 195 (K195) residues within the BBOX1 domain of TRIM24 proteins are those that mediate the TRIM24/H3K27ac interaction and the TRIM24-mediating STAT3 recruitment (Figure 2a). Importantly, these interactions mediate transcription activation in EGFR/EGFRvIII-driven tumorigenesis, stemness and aggressiveness of GBM [83]. Zhang et al. reported that TRIM24 was co-expressed with certain stem-cell markers and its protein levels were markedly increased in glioma stem cells (GSCs) and neural stem cells (NSCs) compared to GBM cells or normal astrocytes [96]. TRIM24 was also interacting with the SOX2 promoter in vitro, thereby mediating its transcription in GSC-containing GBM tumors [96,97]. Importantly, Han et al. treated patient-derived GSC cell lines with two TRIM24 inhibitors (IACS-9571 and dTRIM24), which interact with the TRIM24 bromodomain. The reduction of tumor sphere formation, GSC proliferation, and self-renewal was partially attributed to decreased SOX2 expression [98] (Figure 2a).

TRIM28 (also known as TIF1β or KAP1) is another Group C-VI protein with important implications in gliomas. Like TRIM24, TRIM28 has been recognized as a protein with a versatile role within the malignant cell both as an E3 ligase in the cytoplasm (Figure 2a,b) and as an epigenetic reader and gene co-repressor in the nucleus (Figure 2a,c). Studies of two decades ago, had already provided key evidence for the role of TRIM28 in gliomas. Specifically, Golding et al. reported that ATM inhibition by KU-60019 hindered the phosphorylation of TRIM28, alongside p53 and H2AX, affecting DNA damage pathways and leading to enhanced radiosensitivity in glioblastoma cell lines [99]. At about the same time, TRIM28 phosphorylation was shown to pose an important barrier to DNA double-strand break (DSB) repair within heterochromatin. ATM-dependent phosphorylation of TRIM28 inhibits TRIM28 action and promotes heterochromatic DSB repair and chromatin relaxation via the activation of the CHD3 nucleosome remodeler [100]. Furthermore, a link between *MGMT* promoter-hypermethylation and increased phosphorylation of the DNA Damage Response (DDR) proteins (TRIM28, Chk1, Chk2, and H2AX) was reported in a study comparing veliparib/TMZ versus TMZ-alone treatment [101].

In recent studies, TRIM28 was reported to be differentially expressed in GBM and higher in the classical (CL) GB subtype versus the mesenchymal (MES) subtype. Moreover, studies based on a nanobody-based anti-proteome approach revealed that TRIM28 can be employed as a diagnostic marker for distinguishing glioblastomas from lower-grade gliomas [102]. The effect of the anti-TRIM28 nanobody was more notable on GSC invasion than on differentiated GB cells [82]. Furthermore, TRIM28 was upregulated in GBM-stem-like cells, glioma tissues and cell lines, compared to normal counterparts. TRIM28 expression levels correlated with the tumor grade and presented a marker for poor prognosis. Knockdown of *TRIM28* was shown to abolish glioma cell proliferation in vitro and tumor growth in vivo, further supporting an oncogenic role for TRIM28 (Table 2). Silencing of TRIM28 induced the expression of p21, whereas patients exhibiting the high TRIM28/negative p21 expression combination were of a poor prognosis [103]. Additionally, a study by Peng et al. showed that TRIM28 was upregulated in gliomas (mostly Grade III and IV), and it was correlated with autophagy. Functional assays provided further evidence that TRIM28-mediated glioma cell proliferation was due to TRIM28-induced autophagy [35].

TRIM28 and its epigenetic role as transcriptional co-repressor (Figure 2c) has been well established in cancer [30], including glioma cells. For instance, inhibition of histone methyltransferases SETD8 and G9a was reported to increase radiosensitivity of glioma cells. In particular, loss of H3K9 methylation reduced DNA damage and ATM signaling and, of note, reduced phosphorylation of the KAP1 (TRIM28) repressor [104]. These data highlight the importance of TRIM28 as an epigenetic player (Figure 2c) in abolishing tumor suppressive regulatory programs, thereby exerting its oncogenic potential, conferring, among other things, a resistance to radiotherapy. Another histone methyltransferase, SETDB1, has also been implicated in gliomagenesis [105]. SETDB1 expression was found upregulated in glioma cell lines and glioma tissues compared to a normal brain, and it was positively correlated with tumor grades and histological type. Suppression of SETDB1 was shown to affect cell proliferation, migration, and colony formation of glioma cells [105,106]. Interestingly, SETDB1 is a member of the KAP1 co-repressor complex, and TRIM28 protects SETDB1 from degradation [107].

A recent study demonstrates that TRIM28 is part of a co-repressor complex in glioblastoma. Yu and colleagues investigated the regulatory mechanisms for SIX3, a transcription factor vital for neurogenesis with a bivalent promoter [108]. SIX3 was previously shown to act as a tumor suppressor in glioma cell lines [109]. SIX3 was shown to be regulated by the EGFR-ZNF264 axis. More specifically, EGFR activation leads to *SIX3* promoter DNA methylation through MAPK signaling. Activated ERK binds to ZNF263, abrogating its ubiquitination and stabilization. ZNF263 then binds to the core promoter region of *SIX3* and recruits the TRIM28/HATS/DNMT corepressor complex. TRIM28 is a key element of the co-repressor complex that induces *SIX3* silencing by both the H3K27me3 and DNA methylation at the *SIX3* promoter. Interestingly, the activity of the EGFR-ZNF263 signaling axis was shown to enhance tumorigenicity and be associated with a poor prognosis in glioblastoma [108].

Exciting insights on the epigenetic role of TRIM28 have arisen by multi-omics studies on the differentiation of embryonic stem cells (ESCs) to postmitotic neurons [110]. Bunina et al. reported that genomic rewiring of SOX2-related chromatin networks is a key phenomenon that drives neuronal differentiation. Of note, when researchers evaluated SOX2 interactors, TRIM28 was shown to preferentially bind to SOX2 in ESCs, whereas ADNP and MYEF2 preferentially bound to SOX2 in neurons. A later study showed that SOX2 together with OCT4 induce an immunosuppressive phenotype of GSCs in a BRD/H3k27Ac-dependent manner [111] (Figure 2a–c). The crosstalk of TRIM28 with transcription factors of stemness (Figure 2a) in glioblastoma is further explored in a recent study by Porčnik and colleagues [82]. Specifically, they reported that GBM core versus rim is characterized by an altered cancer stem cell marker profile. Both GSC stemness-related genes (SOX2 and ID1) and MES subtype-related genes (THBS1 and CD44) were enriched in the core of the GBM tumors, along with significantly higher TRIM28 expression. The enrichment of TRIM28 expression was associated with GSCs, homing to the core of the tumor [82]. This finding is in agreement with the earlier observations of higher TRIM28 expression in GSCs compared to GBM cells [102].

TRIM28 forms complexes to exert its versatile roles, not only in the nucleus but also in the cytoplasm (Figure 1). For instance, the melanoma antigen A6 (MAGEA6)/TRIM28 complex is a cancer-specific ubiquitin ligase which targets the tumor suppressor AMP-activated protein kinase (AMPK) for degradation, thus promoting oncogenesis. MAGEA3/6 is activated in response to energy stress and can rewire cancer metabolism through mTOR-dependent cell survival mechanisms. It is thus considered to be a master sensor of cellular energy. AMPK promotes catabolic processes while inhibiting anabolic processes and cell growth to restore energy balance. Depletion of MAGEA3/6 or TRIM28 increases both total and active AMPK levels, leading to the suppression of the mTOR signaling pathway. AMPK accumulation upon *TRIM28* knockdown mediates the metabolic switch from OXPHOS to glycolysis (Figure 2b) [29]. Pan and colleagues reported that MAGEA6 is expressed in human glioma tissues and cells and correlates with AMPKα1 downregulation. Moreover, *MAGEA6* knockdown restored AMPKα1 expression and inhibited glioma cell survival via mTORC1 inactivation as well as glioma xenograft growth [112], while AMPKα1 silencing ameliorated glioma cell death. A later study showed that MAGEA6-AMPK signaling was activated by silencing the long non-coding RNA THOR, which inhibited human glioma cell survival [113]. Lnc-THOR functions via a conserved interaction with insulin-like growth factor 2 mRNA-binding protein 1 (IGF2BP1) and binding to IGF2BP1 was shown to be essential for Lnc-THOR function. Interestingly, IGF2BP1 is upregulated in human glioma tissue and associated with cell proliferation, migration, invasion, and tumor progression. Besides Lnc-THOR, other non-coding RNAs (i.e., miR- 4500, miR-837, miR-506, and LINC00689) have been reported to promote tumorigenesis in gliomas through targeting IGF2BP1 [114,115]. Inhibition of the axis MAGE3/6-TRIM28 and OXPHOS has been also proposed as a strategy for immunotherapy in glioblastoma. Targeting tumor metabolism and the mitochondrion emerges as an attractive therapeutic opportunity for certain subtypes of the disease [116,117].

For the third member of the group C-VI, TRIM33 (TIF1γ, also known as RFG7, PTC7, or Ectodermin), a tumor suppressor potential has been recently suggested in brain tumors [118]. TRIM33 acts as an E3 ubiquitin ligase and targets nuclear β-catenin for degradation. The TRIM33-mediated nuclear β-catenin degradation leads to the suppression of glioma cell proliferation. Furthermore, TRIM33 levels are inversely correlated with β-catenin in GBM patient samples [118]. TRIM33 was shown to interact with β-catenin within the nucleus of GBM cells with a constitutively active EGFRvIII mutant. A negative regulatory loop between TRIM33 and β-catenin for the inactivation of the Wnt pathway upon overstimulation was reported (Figure 2a). Aberrant active Wnt signaling was shown to activate PKCδ, which in turn mediated the phosphorylation of β-catenin at Ser715 and promoted TRIM33 and β-catenin interaction. The consequent degradation of β-catenin inactivated Wnt signaling (Figure 2a). Importantly, the group suggested that TRIM33 may act as a tumor suppressor in GBM since its downregulation and subsequent Wnt pathway constant activation, promoting tumorigenesis in vivo and GBM cell proliferation in vitro. TRIM33 expression leads to accumulation of β-catenin in the cytoplasm and its depletion from the nucleus, and IGFBP2 stabilizes the cytoplasmic β-catenin which is involved in the Oct4 transcripts regulation. IGFBP2 overexpression in GBM cells was shown to regulate TRIM33, β-catenin and Oct4. IGFBP2 and IGFBP2-induced TRIM33 were associated with stemness induction of glioma cells [119]. These findings suggest a novel therapeutic focus area in cancer aiming at aberrant activation of β-catenin [118]. However, TRIM33 may play further roles in the nucleus as a transcriptional regulator (Figure 2a). For example, the EN1 transcription factor regulates neurogenesis-related genes and EN1-bound chromatin complexes are associated with the TRIM33 (among other members of the family Group C-VI) [120]. High expression of EN1 correlates with an increased risk of developing brain metastases in breast cancer patients [120]. Finally, a study profiling diffuse leptomeningeal glioneuronal tumors (DLGNT) revealed fusions of *TRIM33:RAF1* among other genetic lesions leading to aberrant MAPK/ERK signaling [121].

### 4.4. Family Group C-VII: TRIM3 and TRIM32

TRIM3, an NHL- and filamin-domain-containing TRIM protein (Figure 1) seems to exert a tumor suppressor role in glioma cells linked to the control of c-MYC, restoration of asymmetric cell division and attenuation of Notch Nuclear Transport [122,123]. Interestingly, an early study had reported loss of TRIM3 heterozygosity (LOH) via frequent deletions at 11p15.5 in primary human gliomas [124]. Besides TRIM3, the β-globin gene cluster resides in the chromosome region 11p15.5, harboring immunity-related genes, such as IGF2, H19, PHLDA2/TSSC3, and SLC22A18, associated with cancers and gliomas [125].

Another Group C-VII protein, TRIM32 was reported as being upregulated in glioma tissues. Cai et al. showed that overexpression of TRIM32 promotes glioma cell proliferation and confers cell resistance to TMZ [126]. Conversely, knockdown of TRIM32 inhibited glioma cells proliferation in vitro and in vivo and sensitized glioma cells to TMZ in a p53-dependent and -independent manner. This was partially attributed to the TRIM32-mediated apoptosis. TRIM32 interacts with the antiapoptotic proteins BCL-xL and BCL-w, which antagonize the inhibitory effect of TRIM32 knockdown.

### 4.5. Family Group C-VIII: TRIM37

TRIM37 is the single member of the Group C-VIII characterized by a MATH domain (Figure 1). TRIM37 was found to be significantly overexpressed, both at the mRNA and protein level, in glioma tissues and cell lines in contrast with adjacent normal tissues and human astrocytes. Moreover, knockdown of TRIM37 impeded proliferation, migration, and invasiveness of glioma cells. Of importance, TRIM37 downregulation prohibited the activation of the PI3K/Akt pathway, as seen by the decrease in PI3K and Akt phosphorylation (Figure 2a). Thus, Tang et al. suggested that TRIM37 expression might promote glioma aggressive features, such as EMT, by activating the PI3K/Akt axis [81].

### 4.6. Family Group X: TRIM45

TRIM45 is the only member assigned to Group X and is characterized by a Filamin group—like the Group C-VII TRIM proteins—but is missing the NHL domain (Figure 1). TRIM45 is downregulated in glioma tissues compared to a normal brain. TRIM45 expression levels are also inversely correlated with tumor grades, as HGGs (Grade III/IV) were reported to exhibit significant lower levels of TRIM45 than LGGs (Grade I/II). Overexpression and knockdown experiments in GBM cell lines and xenograft models suggested a tumor suppressive role of TRIM45, as its expression attenuated GBM growth. The growth inhibitory effect of TRIM45 relied on activation of apoptotic pathways. Furthermore, TRIM45 was identified as a mediator of transcriptional activity of p53 and induced glioma cell apoptosis in a p53-dependent manner. Moreover, p53 proteasomal degradation was rescued in the presence of TRIM45 and indicated an interaction between the two proteins. Notably, Zhang et al. demonstrated that TRIM45 primarily binds to p53 via its FLMN region, while the CC region might also be acquired for their interaction. Regarding p53 structure, the amino acid sequence 301–393 of its C-terminal domain was shown to be mandatory for its binding to TRIM45. In conclusion, the study revealed that TRIM45 regulates K63-linked polyubiquitination on the C-terminal six lysine residues of p53 via its E3 ligase activity, thereby hindering the subsequent K48-linked polyubiquitination of these residues which would result in p53 degradation [85].

### 4.7. No-Ring Group: TRIM14, TRIM44, and TRIM66

The members of the no-Ring Group are distinct from all other groups C-I–C-XI as they do not bear a Ring-domain and therefore are also called BCC motif-containing TRIM proteins (Figure 1). TRIM14 and TRIM44 share structural similarities, but TRIM44 does not possess SPRY and PRY domains, as opposed to TRIM14 [26,36]. Feng et al. reported that TRIM14 expression was markedly increased in GBM tissues and cell lines compared to a normal brain and human astrocytes, respectively. The TRIM14 expression levels correlated positively with the glioma malignancy grade and were indicative of a poor clinical outcome. Through loss-of-function and gain-of-function assays, it was shown that TRIM14 induced EMT, migration, and proliferation of glioblastoma cells. A connection between TRIM14 and the EMT-promoting transcription factor ZEB2 was reported, as TRIM13 can obstruct the poly-ubiquitylation of ZEB2, and hence its proteasomal degradation, by impeding the function of the F-Box protein 45 (FBXO45) E3 ubiquitin ligase. In vivo experiments showed that GBM cells were significantly less invasive in *TRIM14*-knockdown mice, due to the interplay between TRIM14-ZEB2. These findings were also confirmed in GBM samples, where TRIM14 levels correlated with ZEB2 levels [127]. In a later study, Deng et al. reported that circ_0005198 and TRIM14 expression were highly expressed in glioma tissues and TMZ-resistant glioma cell lines compared to a normal brain and astrocytes, whereas miR-198 levels were significantly lower. They provided evidence that circ_0005198 functions as a cytoplasmic sponge for miR-198 via a shared sequence in its 3′-UTR and that the negative regulation of miR-198 was linked to TMZ-resistance. TRIM14 3′UTR was also identified as a target of miR-198, and an oncogenic role for TRIM14 has been suggested since high levels of TRIM14 were conferring TMZ-resistance [128].

Regarding TRIM44, studies in GBM cell lines showed that it is targeted by miR-101-3p through binding to *TRIM44* 3′-UTR which blocks its transcription. Of note, miR-101-3p expression in GBM cell lines was hampering proliferation, colony formation, migratory and invasive properties of glioma cells, and suppressing EMT via the Wnt/β-catenin pathway (Figure 2a). On the contrary, TRIM44 expression was found to enhance the aforementioned processes and thereby an oncogenic role for TRIM44 was proposed [129] (Table 2). One year later, Zhou et al. showed that high TRIM44 expression correlates with poor survival of glioma patients. TRIM44 expression was found increased in GBM and GSC cell lines compared to normal human astrocytes and *TRIM44* knockdown resulted in glioma cell proliferation and migration inhibition, downregulation of EMT marker genes and activation of apoptotic pathways. Furthermore, *TRIM44* silencing led to decreased phosphorylation of Akt (Figure 2a) and a subsequent increase in p21/p27 levels, which are known to cause cell cycle arrest [130].

Another recent study by Song et al. showed that another no-Ring TRIM protein, TRIM66 (Figure 1) was highly expressed in gliomas compared to normal brain tissues and in a tumor-grade-associated manner (significantly upregulated at Grade III gliomas and GBM). This shows that TRIM66 can exert an oncogenic potential since in vitro and in vivo assays showed that TRIM66 induces glioma cell proliferation, migration, and tumor growth. Conversely, TRIM66-silencing was activating apoptotic pathways. In addition, TRIM66 appears to regulate the metabolic potential of glioma cells, since its expression affected ATP levels and glucose uptake. TRIM66 also impacts c-MYC and GLUT3 protein expression levels. Mechanistically, TRIM66-induced upregulation of GLUT3 protein was shown to be a result of the binding of c-MYC on SLC2A3/GLUT3 promoters in glioma cells [131].

**Table 2:** Main TRIM protein family members implicated in gliomas.

Group	TRIM	Expression and Function	Role *	References
**C-IV**	TRIM11	↑ in HGG and glioma-derived GSCs aggressive phenotype -poor prognostic marker marker for GSC improved detection EGFR/MAPK signaling	ONC	[86]
	TRIM17	↓ in gliomas vs. normal tissue inverse correlation with tumor grade ↓ glioma cell proliferation	TS	[87]
	TRIM21	↑ in gliomas/correlation with tumor grade poor prognostic marker (OS and PFS) potential prognostic value in hGGs including GBM ↑ GBM patients with IDH1wt or non-G-CIMP > IDH1mut or G-CIMP ↑ in patients with unmethylated MGMT promoter ↑glioma cell proliferation and migration ↑ glioma progression ⇒ TRIM21-mediated cellular senescence via p53/p21 ↑ treatment resistance to TMZ	ONC	[88]
	TRIM22	↑ in primary hGGs > lGGs > normal ↑ tumor growth and glioma cell proliferation NF-κΒ signaling ⇒ stability of IκBα	ONC	[89]
	TRIM47	↑ in gliomas vs. normal tissue poor prognostic marker ↑ in GBM > lGGs > normal, correlation with tumor grade Silencing ⇒ ↓ proliferation, invasiveness, migration, EMT markers, tumor growth Wnt/b-catenin pathway	ONC	[90,91]
	TRIM65	↓ in gliomas ⇒ regulated by miR-1281 and LINC01857 induced TRIM65 expression in gliomas by restraining miR-1281 ↑ LINC01857 levels in glioma cell lines ⇒ ↑ glioma cell proliferation, migration and invasiveness	UNCLEAR	[92]
**C-V**	TRIM8	frequent hemizygous deletion (88%) in GBMs GBM tissues and cell lines vs. normal ⇒ similar expression levels but different subcellular localization GBM ⇒ in the nucleus vs. normal (cytoplasmic) correlation with stemness marker positive feedback loop between TRIM8 and STAT3, ⇒ GBM neurosphere stemness regulation STAT3 ⇒ ↑ TRIM8 expression either directly or indirectly via c-MYC and OCT1	DUAL	[93,132]
	TRIM31	↑ in glioma tissues and cell lines ↑ glioma cell proliferation and invasiveness ↑ NF-κB signaling pathway ⇒ p65 translocation to the nucleus and IκBα phosphorylation poor prognostic marker in hGGs Akt signaling pathway ⇒ oncogenesis	ONC	[94,95,133]
**C-VI**	TRIM24	↑ in hGGs, GSCs and NSCs of GBM correlation with stemness markers in GBM poor prognostic marker in GBM (OS) and for chemo-treated glioma patients (OS and PFS) required for EGFR activation and STAT3 recruitment/stabilization ↑ GBM growth via Akt phosphorylation and regulation of PI3KCA ↑ TMZ treatment resistance via NF-κB signaling ↑ GBM tumorigenesis, stemness and aggressiveness via TRIM24/H3K27ac marks interactions ⇒ EGFR/EGFRvIII its expression regulated by STAT3 SOX2 promoter interaction in GSC-containing GBM samples ACS-9571 and dTRIM24 inhibitors ⇒ ↓ SOX2 expression and ↓ tumorigenicity in GSCs	ONC	[83,84,98]
	TRIM28	↑ GBM samples ↑ ↑ classical > ↑ mesenchymal subtype correlation with tumor grade poor prognostic marker (OS) TRIM28 downregulation ⇒ ↑ p21 TRIM28high/p21neg ⇒ poor prognosis ↑ TRIM28 ⇒ ↑ autophagy TRIM28/HATS/DNMT complex ⇒ ↓ SIX3 ↑ EGFR-ZNF263 signaling ⇒ ↑ tumorigenicity MAGEA3/6-TRIM28 complex ⇒ ↓ AMPK ⇒ metabolic switch OXPHOS/glycolysis	ONC	[35,82,103,108,112,134]
	TRIM33	targets nuclear β-catenin for degradation ⇒ ↓ glioma cell proliferation inverse correlation with β-catenin in GBM induced by IGFBP2 ⇒ ↑ TRIM33 ⇒ ↑ cytoplasmic β-catenin and ↓ nuclear β-catenin ↑ IGFBP2 overexpression in GBM ⇒ ↑ TRIM33 ⇒ ↑ stemness induction fusions of TRIM33:RAF1 in DLGNT ⇒ aberrant MAPK/ERK signaling	TS	[118,119,121]
**C-VII**	TRIM3	altered genomic dosage of TRIM3 was detected in gliomas, including homozygous deletions of TRIM3		[124]
	TRIM32	↑ in gliomas ↑ cell proliferation ⇒ ↑TMZ resistance p53-dependent and -independent pathways interaction with antiapoptotic proteins BCL-xL and BCL-w		[126]
**C-VIII**	TRIM37	↑ in glioma tissues and cell lines vs. normal tissues and human astrocytes ↑ proliferation, migration/invasion/EMT ↑ glioma aggressiveness via PI3K/Akt axis activation	ONC	[81]
**C-X**	TRIM45	↓ in gliomas inverse correlation with tumor grade tumor suppressive function in gliomas ⇒ activating apoptotic pathways in a p53-dependent manner ↓ ubiquitination via its ligase activity	TS	[85]
**no-Ring**	TRIM14	↑ GBM tissues and cell lines correlation with tumor grade poor prognostic marker (OS) ↑ proliferation and ↑ migration ↑ EMT by blocking ZEB2 proteasomal degradation	ONC	[127]
	TRIM44	↑ proliferation ↑ migration ↑ invasiveness of glioma cells EMT mediation via the Wnt/b-catenin pathway poor prognostic marker in glioma (OS) ↑ in GBM and GSC vs. normal TRIM44 inhibition by miR-101-3p in GBM cell lines Silencing ⇒ ↓ phospho-Akt and ↑ p21/p27 ⇒ cell cycle arrest	ONC	[129,130]
	TRIM66	↑ in glioma tissues vs. normal brain correlation with tumor grade ↑ cell proliferation, migration and tumor growth ↑ ATP levels and glucose uptake ↑ c-MYC and GLUT3 expression	ONC	[131]

* Role in Gliomas: ONC—Oncogene, TS—Tumor Suppressor. OS—Overall Survival, PFS—Progression Free Survival.

## 5. Conclusions

TRIM proteins are without doubt vital for the conservation of cellular homeostasis. These multidomain-containing proteins play a multifaceted role in gene expression regulation and cell signaling repertoire, thus affecting a wide range of processes, such as DNA repair, autophagy, and apoptosis. The complexity of their biological nature can be depicted by their contribution in both physiological and pathological conditions depending on the context. Regarding the nervous system, TRIM proteins may act as guardians by confining inflammation and mediating neural differentiation programs, thus ensuring proper brain development. However, several members of the family (TRIM11, TRIM17, TRIM21, TRIM22, TRIM47, TRIM65, TRIM8, TRIM31, TRIM24, TRIM28, TRIM33, TRIM37, TRIM45, TRIM14, TRIM44, and TRIM3) seem to be implicated either as oncogenes or tumor-suppressors in the pathogenesis of gliomas.

The dual role of TRIMs in these CNS malignancies, which is extensively argued in this review, highlights their potential as prognostic biomarkers and therapeutic targets. As mentioned before, TRIM24, TRIM47, TRIM44, TRIM31, TRIM14, TRIM21, and TRIM28 have exhibited significant prognostic value regarding OS and/or PFS of glioma patients. TRIMs may also prove useful as targets in the therapy of gliomas. For instance, regarding TRIM24, which functions as an oncogenic factor in gliomas, four inhibitors (Compound 34, IACS-6558, IACS-9571, and dTRIM24) have been developed so far. Inhibitor dTRIM24 induces the degradation of the TRIM24 protein, while the rest impede the protein’s function by targeting its bromodomain [98]. Therefore, it is of great importance to further unveil the role of TRIM proteins in gliomagenesis and exploit their potential as prognostic and therapeutic tools.

## Abbreviations

ARF	ADP ribosylation factor-like
BioID	Biotin Identification
BRD	Bromodomain
CC	Coiled-Coil
*CCND1*	Cyclin D1
CL	Classical
CNS	Central Nervous System
COS	C-terminal subgroup one signature
DDR	DNA damage response
DEGs	Differentially Expressed Genes
DLGNT	Diffuse Leptomeningeal Glioneuronal Tumors
EE	Epileptic Encephalopathy
ESCs	Embryonic Stem Cells
EGFRvIII	EGF receptor vIII
EMT	Endothelial to Mesenchymal Transition
ERVs	Endogenous retroviruses
FBXO45	F-Box protein 45
FN3	Fibronectin type III motif
GBM	Glioblastoma
GSCs	Glioma-derived stem cells
H3	Histone 3
H3K23ac	H3 lysine 23 acetylation
HB-EGF	Heparin-binding EGF-like growth factor
HFD	High Fat Diet
HGGs	High Grade Gliomas
ID1	Inhibitor of DNA binding 1
IRS1	Insulin Receptor Substrate-1
K195	Lysine 195
KAP1	KRAB-associated protein-1 (TRIM28)
KEAP1	Kelch-like ECH- Associated Protein 1
LGGs	Low Grade Gliomas
LGMDR8	Limb–Girdle Muscular Dystrophy R8
lncRNAs	Long Non-Coding RNAs
LOH	Loss of Heterozygosity
MAPK	Mitogen-Activated Protein Kinase
MATH	Meprin And Tumor-necrosis factor receptor-associated factor Homology
MES	Mesenchymal
MG53	Mitsugumin 53
MGMT	*O6-methylguanine-DNA methyltransferase*
NPCs	Neural Progenitor Cells
NSCs	Neural Stem Cells
OGD/R	Oxygen-Glucose Deprivation/Reperfusion
OPCs	Oligodendrocyte Progenitor Cells
OPTN	Optineurin
OS	Overall Survival
OSKM	OCT4, SOX2, KLF4, and c-MYC
PFS	Progression Free Survival
PHD	Plant Homeodomain
PSD	Post Synaptic Density
R193	arginine 193
RBCC	RING-B-box-Coiled-Coil
RNAi	RNA interference
shRNAs	Short Hairpin RNAs
SLE	Systemic Lupus Erythematosus
TEs	Transposable Elements
TIF1	Transcriptional Intermediary Factor 1
TIF1γ	Transcriptional Intermediary Factor 1 γ
TMZ	Temozolomide
TRIM	Tripartite Motif-containing
